# Consensus on addressing HIV-related stigma and achieving the societal enabler targets using an adapted Delphi process

**DOI:** 10.1136/bmjopen-2024-092516

**Published:** 2025-08-12

**Authors:** Kate Molesworth, Sbongile Nkosi, Salvador Camacho, Georgina Caswell, Simone Salem, Stefan Baral, Adeeba Kamarulzaman, Laurel Sprague, Lucy Stackpool-Moore

**Affiliations:** 1 Swiss Tropical and Public Health Institute, University of Basel, Basel, Switzerland; 2 Global Network of People Living with HIV (GNP+), Johannesburg, South Africa; 3 Swiss Tropical and Public Health Institute, Allschwil, Switzerland; 4 Global Network of People Living with HIV (GNP+), Cape Town, South Africa; 5 Human Rights Team Equality and Rights for All Department, UNAIDS, Geneva, Switzerland; 6 Johns Hopkins University Bloomberg School of Public Health, Baltimore, Maryland, USA; 7 Monash University Malaysia, Bandar Sunway, Malaysia; 8 International AIDS Society, Geneva, Switzerland; 9 UNAIDS, Geneve, Switzerland; 10 Watipa, Sawtell, New South Wales, Australia

**Keywords:** Health policy, HIV & AIDS, PUBLIC HEALTH

## Abstract

**Abstract:**

**Objectives:**

To seek consensus among global experts on concepts, measures and approaches to guide national and global action to address HIV-related stigma and formulate a call to action. This outlines priorities to unite actors in more effectively responding to and resourcing efforts to address HIV-related stigma.

**Design:**

An adapted Delphi consensus-building process using two rounds of online questionnaires.

**Setting:**

Online questionnaires sent to a global expert panel.

**Participants:**

50 global experts on HIV-related stigma and discrimination representing sectors including civil society, people living with HIV and key populations, research and academia, clinical practice, law, non-profit organisations, the United Nations, and policy and donor organisations.

**Results:**

The panel reached consensus on 55 points relating to the 12 broad themes extracted from the evidence base. These comprised the importance of addressing HIV-related stigma at scale; HIV-related stigma terms and definitions; Frameworks; Programming and approaches; Community leadership in HIV-related stigma-reduction implementation; Intersectional stigma and discrimination; Stigma and discrimination measures and assessment scales; Monitoring and evaluation; Stakeholder and community participation in monitoring and evaluation; Knowledge gaps and research needs; Funding and Commitment calls. From these, a consensus statement and call to action were formulated on priorities for strong political and financial commitments by all countries to reduce and mitigate HIV-related stigma and achieve global HIV targets adopted in 2021.

**Conclusions:**

This study illustrated that global experts across sectors consider that action is needed to support the three critical enablers of the HIV response—society, systems and services—to ensure that HIV services are non-discriminatory and person-centred. The importance of attention and action to reduce stigma is critical in the current geopolitical and funding crisis affecting HIV and global health.

**STRENGTHS AND LIMITATIONS OF THIS STUDY**

- This study enabled global experts to review the evidence base, form a consensus on its gaps and formulate a consolidated call for action to address HIV-related stigma.
- Reaching consensus on a large number of themes and points of evidence was complex and required a methodological adaptation that deviated from traditional Delphi methodology often used in consensus-building.
- As agreement was very high on many of the first 100 survey questions, the consensus level was set after the results emerged, which could be interpreted as the researchers controlling what should and should not be included.
- The study was conducted in English and was shaped by the evidence reported in English, which limited the linguistic inclusion of the expert panel and scope of the consensus process.

## Introduction

 There has been strong global commitment to eliminate HIV-related stigma; however, the recent geopolitical context has jeopardised gains, and future progress is uncertain. Modelling has indicated that funding reductions in 2025 (such as changes to the United States Agency for International Development and the President’s Emergency Plan for AIDS Relief) could significantly reverse progress in the HIV response by 2030, disproportionately affecting sub-Saharan African countries and key and vulnerable populations.^1^ HIV-related stigma and discrimination, including within healthcare settings, still constitute significant barriers to HIV responses around the world, and in the current funding context will continue to hamper the achievement of national and global targets.

Societal and legal barriers hinder quality HIV prevention, care, treatment and support services and must be removed. At the high-level meeting on HIV and AIDS in June 2021, United Nations Member States adopted a political declaration that expanded the UNAIDS targets to 10 which included 3 societal-enabler global targets that had been set to be achieved by 2025.^2^ In particular, this included a target of less than 10% of people living with HIV and key populations experiencing stigma and discrimination. Mathematical modelling indicates that without addressing stigma, we will have missed the UNAIDS 2025 targets to end AIDS as a public health threat by 2030.^3^ These targets are defined as 95% of all people living with HIV to know their HIV status, 95% of all people diagnosed with HIV to receive sustained antiretroviral therapy (ART), and 95% of all people receiving ART to have viral suppression by 2025.^2^ A focus beyond viral suppression and clinical indicators to include quality of life of people living with HIV, sometimes referred to as the fourth ‘90’, has also been considered and been included in many contexts.^4^ The current geopolitical context and threats to HIV funding has further impeded progress in these areas. 

Achieving the UNAIDS 2025 clinical and societal-enabler targets would have required concerted efforts to remove barriers to progress. Recognising community leadership in addressing stigma and discrimination and enabling quality and stigma-free services will contribute to achieving UNAIDS’ 30-80-60 targets. These are defined in the Global AIDS Strategy: 30% of testing and treatment services to be delivered by community-led organisations; 80% of service delivery for HIV prevention programmes for key populations and women and girls to be delivered by their respective community-led organisations; and 60% of the programmes to support the achievement of societal enablers to be delivered by community-led organisations.^5^

A recent systematic review to identify frameworks and measures aimed at understanding or assessing internalised stigma, and stigma and discrimination in healthcare and in law and policy found wide variations in the frameworks and measures used.^6^ Analysis of stigma interventions to date shows that some researchers and practitioners use different definitions of HIV-related stigma and that there is a general lack of consensus on what stigma actually is and, consequently, how to address it.^7^ This situation hampers learning across interventions. Other recent systematic reviews have called for greater attention to strengthening and expanding the development and evaluation of multilevel stigma interventions^8^ and testing structural interventions and approaches that challenge the systems of power and oppression.^9^ Given the lack of agreement on multiple and intersecting elements relating to HIV-related stigma, we undertook a global process in 2022 to seek consensus among experts on key ideas, concepts, measures and approaches to guide more cohesive responses to HIV-related stigma.

## Methods

### Study design and steering

IAS—the International AIDS Society—convened a steering group of researchers, implementers and policy makers from the broader Global Partnership for Action to Eliminate all Forms of HIV-Related Stigma and Discrimination. The Global Partnership is made up of representatives of the Global Network of People Living with HIV, the United Nations Development Programme, the United Nations Entity for Gender Equality and the Empowerment of Women, the Global Fund to Fight AIDS, Tuberculosis and Malaria, the US Centers for Disease Control and Prevention, and the UNAIDS Secretariat, with leadership and technical support from the Nongovernmental Organization delegation to the Joint United Nations Programme on HIV/AIDS (UNAIDS) Programme Coordination Board.^10^ A list of the country, affiliation and title of each steering group member is set out in online supplemental annex 1. None of these individuals participated in responding to the surveys.

### Consensus-building survey design

A two-phase consensus-building survey methodology was developed as an adaptation of the multistage Delphi process.^11^ This was chosen as a rapid adaptation of an established consensus-building tool to facilitate structured group communication to gather wide expert opinions on complex issues and work methodically to develop a consensus statement on HIV-related stigma. Our method deviated from traditional Delphi methodology in that the first round was designed to obtain consensus regarding the importance that expert panellists gave to evidence relating to concepts, measures and approaches to HIV-related stigma, while the second round was designed to build consensus on prioritising action that the panel considered to be necessary.

Identification of literature to inform the design of the consensus-building survey was conducted through two approaches to ensure the inclusion of relevant peer-reviewed studies, as well as the most relevant non-peer reviewed publications and unpublished data. The first was an IAS-commissioned systematic review, carried out by the University of Southern California, of peer-reviewed and published evidence on HIV-related stigma frameworks, measures and mitigation of HIV-related internalised stigma, and HIV-related stigma and discrimination in healthcare and in laws and policies.^6^ Additionally, the steering group recommended a list of policy and guidance documents, further systematic reviews on interventions to reduce HIV-related stigma and discrimination, and ‘grey’ literature capturing community experience and commentaries. These were the primary sources of evidence used to inform the questions asked in the consensus-building process. Online supplemental annex 3 includes the literature sources informing the consensus points.^36 8 12^,^24^

Thematic categories and specific evidence emerging from the literature review were presented to the steering group at a workshop to form the basis for priority consensus-seeking in the first round questionnaire. An initial list of 100 evidence points for prioritisation by panellists was agreed by the steering group. These were refined into the first round of questions within an online questionnaire (online supplemental annex 4) that was developed by the Swiss TPH researchers and then refined and agreed by the steering group. Questions were presented as short summaries of evidence extracted from the published literature review^6^ and key findings from the grey literature that panellists were asked to rate in terms of importance using a 5-point Likert scale comprising simple choices such as ‘strongly agree’, ‘agree’, ‘undecided’, ‘disagree’ and ‘strongly disagree’, detailed in online supplemental annex 4. Additionally, the questionnaire provided space for panellists to provide additional comments in their own words.

In order to work with the prevailing consensus on a very broad range of topics, we did not preset a consensus percentage, but opted to allow the results to be described in terms of the strength of consensus expressed throughout the two survey rounds. No pilot round was conducted; instead, questionnaires were reviewed by the steering committee and adjusted according to feedback.

### Expert panel recruitment and survey implementation

The Swiss Tropical and Public Health Institute was commissioned to design and implement the survey process in close collaboration with the steering group. The process was conducted virtually, in English, using online questionnaires with an expert panel representing Africa and the Middle East, Western, Central and Eastern Europe, Asia and the Pacific, and North and Latin America and the Caribbean. Links to the first survey were sent to panellists in late October 2022. Participants registered their consent by following invitation links and were free to opt out of any individual questions they did not wish to answer.

The panel of experts was selected via the steering group network of stakeholder organisations and comprised specialists on HIV-related stigma and recruited by email. 50 individuals agreed to participate in the consensus-building process, representing sectors that included civil society, people living with HIV and key populations, research and academia, clinical practice, law, non-profit organisations, the United Nations, and policy and donor organisations An anonymised list of the country, affiliation and title of each panel member is set out in online supplemental annex 2. Panel members were neither reimbursed, given expenses nor in any way offered incentives for their participation. It should be noted that in the anonymous survey responses, panel members were asked to self-identify the sector in which they worked and some selected more than one sector. Patients and the public were not involved in this study.

### Data collection

The finalised first round of consensus-building questions was formatted within the EVASYS survey management software. This provided each panellist with an individual access code and protected anonymity,^25^ which allowed the experts to express their opinions freely, encouraged openness and avoided peer pressure. Guidance and an invitation to participate in the survey were distributed by an introductory email to individual panellists, setting out definitions of terms and providing them with an individual link.

Responses were slow and a number of panellists required technical support in accessing the first survey, and multiple reminders and extensions were made to the submission date in order to support busy participants to engage. Data were analysed and first round quantitative summary reports were distributed to panellists in January 2023, followed by the second survey in February.

### Data analysis

Data generated by the extensive first-round questionnaire were analysed and summarised in a report using simple, descriptive statistics that was shared with the steering group and the expert panellists. In this way, all participants received a full and anonymous record of what other experts’ opinions were, regarding the importance panellists gave to evidence relating to concepts, measures and approaches to HIV-related stigma. The summary report of the first round informed and shaped the second round, which was implemented to determine consensus points elaborated from the expert panel’s responses to the first round of questions. The high level of consensus emerging from the first round of questions led us to define a preliminary consensus of 98%–100% of respondents agreeing on all degrees of agreement (‘strongly agree’ and ‘agree’) on the 5-point Likert scales used. It also informed the research team that only one further consensus-building round of questions was required.

Having determined initial consensus regarding the evidence relating to HIV-related stigma, the second round of the survey, rather than repeating questions in the way that the Delphi process does, was designed to focus on determining consensus on prioritising action that the panel considered needed to be taken on the most important evidence to achieve societal enabler targets at scale. Thrity-one questions were presented to the panel in the second survey round. These comprised questions on the merits of calls for action on the themes that had already achieved 98%–100% consensus in round 1, together with some refined questions to further explore the themes that achieved consensus of between 95% and 97% in round 1. The results of this study are reported in accordance with the ACCORD checklist.

### Patient and public involvement

None.

## Results

### Consensus-building survey response rate

**Figure 1:**
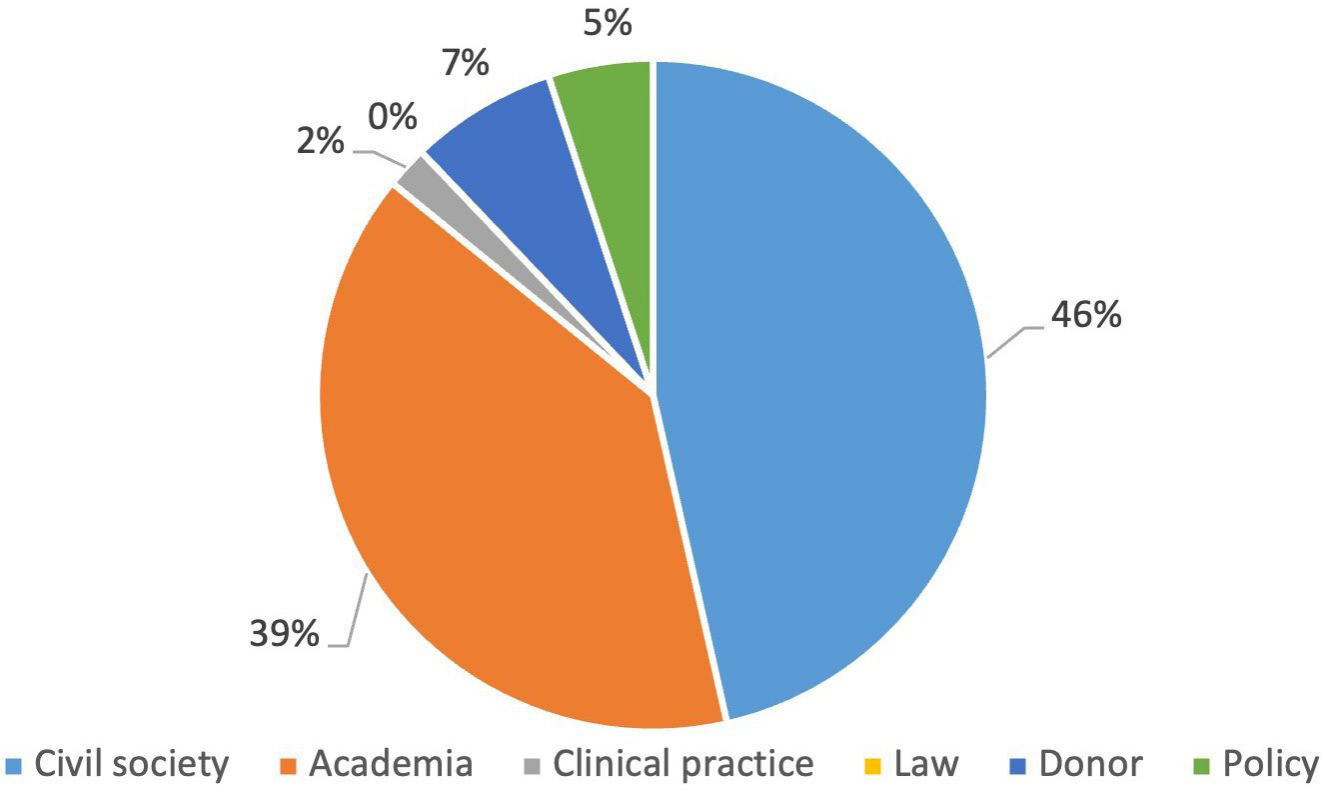
Stakeholders who responded to round 1.

In round 1, there was a response rate of 88%: 44 of the 50 panel members shared their expertise by submitting responses to the survey. The majority self-identified as being part of civil society (figure 1). Details of civil society organisation represented are set out in online supplemental annex 2.

**Figure 2:**
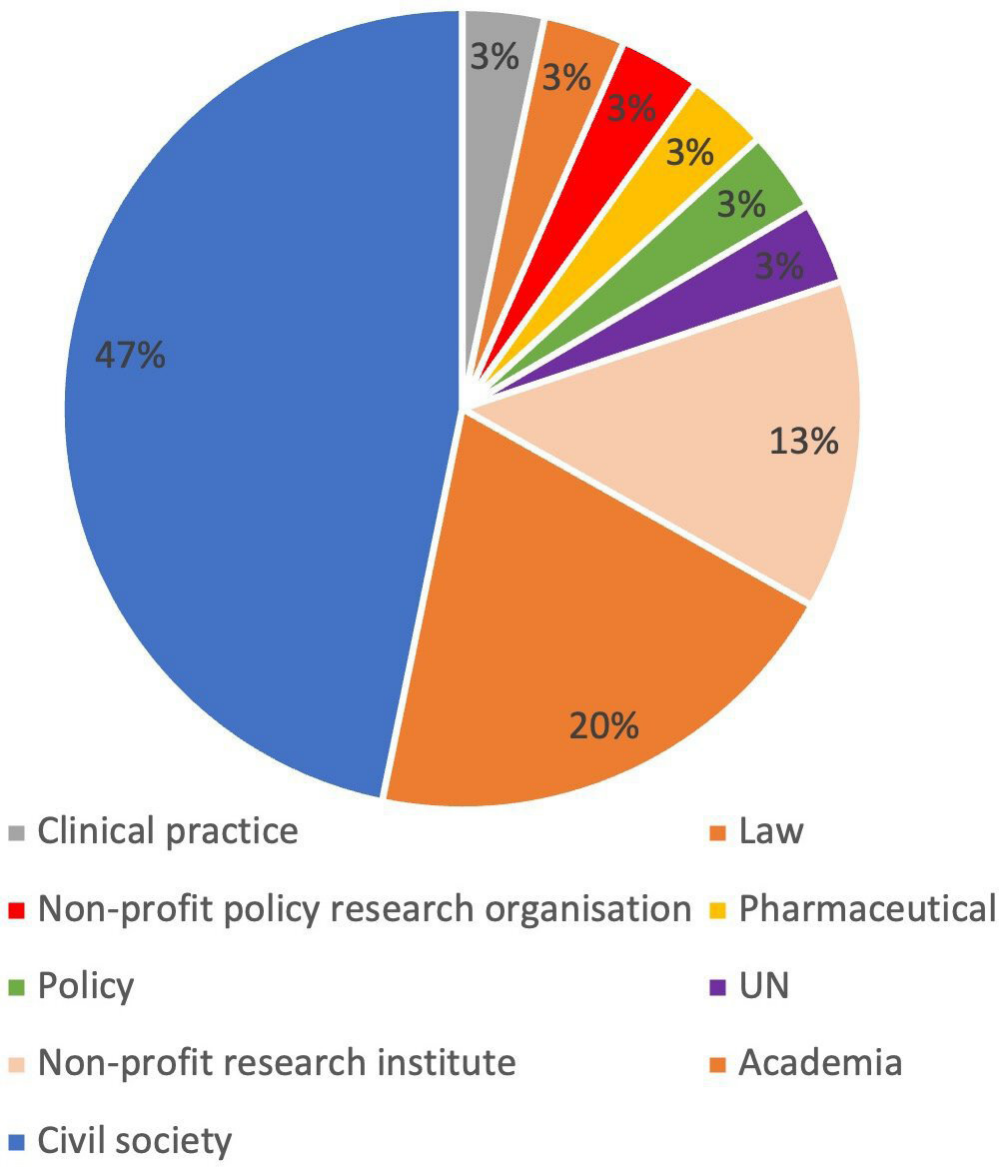
Stakeholders who responded to survey round 2. UN, United Nations.

In round 2, the response rate was 60%, with 30 of the 50 panel members responding (figure 2). This pattern may reflect the fact that both survey rounds were distributed to all panellists, regardless of whether they had engaged in the first round.

### Consensus reached in survey round 1

In the first round, consensus data were generated on more than 100 points of evidence on HIV-related stigma that were presented to panellists. (The original questions and Likert scales are set out in online supplemental annex 4). Overall, there was a very high level of agreement: seven points generated agreement of less than 69%, and 93 questions achieved consensus of 70%–100%. Of these, 44 points reached consensus of 98%–100% on the aggregate of all degrees of firm agreement on the 5-point Likert scales used (eg, ‘very important/important/moderately important’ vs ‘slightly important’ and ‘unimportant’). The results, set out in table 1, show variation in the number of panellists responding to each question, most likely reflecting differences in thematic interest and knowledge among the expert panel.

**Table 1:** Consensus points (98%–100%) on reducing HIV-related stigma at scale following two survey rounds

Theme and consensus points	Survey round	% agreement (on aggregate of all degrees of agreement on the Likert scale)	N*
1. The importance of addressing HIV-related stigma at scale			
1.1. It is important to understand how stigma and discrimination are being experienced.	1	100	43
1.2. It is important to measure stigma in a systematic and thorough manner in order to reach global HIV targets.	1	100	42
2. HIV-related stigma terms and definitions			
2.1. It is important to achieve consensus on definitions and use of stigma-related language.	2	97	30
2.2. Achieving consensus on definitions would enable comparability, cross-setting learning and efforts to assess progress towards global targets.	1	100	43
3. Frameworks			
3.1. Conceptual frameworks are useful in research, real-world intervention development and policy on health-related stigmas.	1	98	43
3.2. Frameworks based on underlying stigmatisation processes and how they manifest should be more frequently used in research, intervention development and policy on health-related stigmas.	1	100	41
4. Programming and approaches			
4.1. It is important for all preservice and in-service healthcare providers to be trained on HIV, human rights, key populations, stigma reduction, non-discrimination, gender sensitisation and ethics.	1	100	44
4.2. Support is needed to strengthen skills and create spaces for diverse representatives of communities most affected by HIV-related stigma to meaningfully engage, influence, advocate and participate in decision-making for programme development in different countries.	1	98	43
4.3. The efforts of the Global Partnership are important in bringing together different stakeholders at country level to develop plans to tackle stigma, led by government and civil society.	1	100	41
5. Community leadership in HIV-related stigma-reduction implementation			
5.1. A combined community-led approach to providing education, counselling, facilitating access to an HIV specialist and engaging a support person should be scaled up globally within stigma-reduction programming.	1	100	43
5.2. It is important that community-led approaches are well documented.	1	100	42
5.3. Standard reporting guidelines should be developed on community engagement in HIV-related stigma reduction implementation.	1	100	43
5.4. The limited involvement of communities in all stages of investment decisions threatens the success of stigma-reduction efforts.	1	100	43
6. Intersectional stigma and discrimination			
6.1. Interventions should endeavour to target a combination of structural-level and individual-level risks and resilience to tackle internalised stigma.	1	100	42
6.2. More must be done to protect people who belong to more than one marginalised group from violence, but such laws must be actively enforced, and reporting systems must be available to report abuse and seek redress.	1	100	43
6.3. Synergistic attention is required in the areas of internalised stigma and stigma and discrimination within healthcare settings.	1	98	41
6.4. Do you agree that a stronger focus is needed on policy-level interventions to address stigma at the institutional and structural levels?	1	100	41
7. HIV-related stigma measures and assessment scales			
7.1. It is important for researchers and programmers to use validated measures for monitoring and evaluation of stigma-reduction and discrimination-reduction efforts.	1	100	42
7.2. It is important for researchers and programmers to use community-led measures for monitoring and evaluation of stigma-reduction and discrimination-reduction efforts.	1	100	41
7.3. It is important to adapt existing standardised stigma instruments/measures to specific cultural contexts.	1	100	42
7.4. The Stigma Index 2.0, designed by the community for the community, measures multiple types of stigmas and should be better represented in the published literature.	1	100	35
7.5. It would be helpful to have guidance on local adaptation of measures.	1	100	40
7.6. Additional effort is needed to measure and evaluate the implications of discriminatory laws and the mechanisms of their impact at the individual and health systems levels.	1	98	40
7.7. It is important that community organisations implementing the Stigma Index are supported to disseminate their findings in the peer-reviewed literature (eg, through training about how the data can be analysed/interpreted in different settings).	1	100	41
8. Monitoring and evaluation (M&E)			
8.1. Many countries do not have a robust M&E approach to capture stigma and discrimination—they should be supported to develop one.	2	97	30
9. Stakeholder and community participation in M&E			
9.1. Experiences of stigma should be monitored by community-led organisations.	2	100	30
9.2. Efforts must be scaled up to enable communities to monitor experiences of stigma, advocate for change as needed, and engage and lead in programme and policy development.	2	97	30
9.3. Community-led efforts must be increased to monitor stigma, discrimination and rights violations.	1	98	41
10. Knowledge gaps and research needs			
10.1. It is important that investment is made to establish a robust evidence base across a range of settings and diverse populations of promising interventions and processes to support stigma reduction.	1	100	41
10.2. There is a need to develop and evaluate more diverse approaches to the effectiveness of stigma-reduction and discrimination-reduction efforts.	1	100	40
10.3. More community-led research is needed about, by and for diverse communities of people living with and affected by HIV, including young people in low- and middle-income countries.	2	97	30
10.4. Efforts should be stepped up to strengthen the evidence and knowledge base on stigma and discrimination in law and policy, especially understanding and responding to the extent to which laws are understood in different settings.	2	97	30
10.5. Stigma-reduction efforts globally would be better served by including broader outcomes, such as well-being, mental health, quality of life and flourishing.	2	97	30
10.6. Research is needed to inform the knowledge gap on the measurement and evaluation of the effectiveness of interventions to address internalised stigma.	1	100	42
10.7. It is important that research be conducted to provide data to support future funding investment in the large number of societal enabling approaches that have been piloted and found to positively influence the effectiveness of HIV services.	1	99	40
11. Funding to address HIV-related stigma			
11.1. It would be useful to regularly review the global funding landscape situation in relation to stigma.	1	100	42
11.2. It would be useful to review and coordinate national funding landscapes in relation to stigma.	1	100	40
11.3. It is important that organisations should track and report their stigma-reduction investments.	1	100	36
11.4. It is important to separately track investments in HIV-related stigma and discrimination within broader HIV investments.	1	100	41
11.5. It is important to rectify the global funding landscape that is insufficient to meet societal enabler targets.	1	100	40
11.6. It is important for major donors to obtain guidance to incorporate due attention to stigma within investments and specific grant allocations.	1	100	39
11.7. It is important for major donors to ensure that diverse and inclusive community engagement is considered to guide investment priorities and/or dissemination of results from investments.	1	100	40
11.8. It is important for major donors to advocate for and convene other funders to enhance investment in stigma-reduction and discrimination-reduction efforts.	1	100	38
11.9. Efforts are needed to explore opportunities for long-term stigma-reduction and discrimination-reduction investments.	1	100	40
11.10. Means must be determined to provide flexible funding mechanisms that could respond to emerging critical issues.	1	100	40
11.11. Efforts are needed to integrate stigma and discrimination reduction within investments and strengthen tracking and reporting.	2	97	30
12. Commitment calls			
12.1. For countries to make concerted efforts to achieve UNAIDS 95-95-95 targets for HIV testing, treatment and viral suppression rates by 2030, commitment is called for to address HIV-related stigma, remove societal and legal impediments to HIV service, scale up treatment and prevention, and improve social conditions.	1	100	43
12.2. Strong political and financial commitment is called for to remove the societal and legal impediments that inhibit quality HIV prevention, care, treatment and support services. This is essential for countries to reach their new societal enabler global targets by 2025.	1	100	42
12.3. Countries are called on to commit to specific goals, such as removing legal environments that impede HIV services, and ensuring that no more than 10% of people living with HIV and people in key populations experience stigma and discrimination.	1	100	41
12.4. High-level commitment is called for to scale up and improve the quality of programmes to reduce human rights-related barriers to HIV services by mainstreaming lessons learnt from the Breaking Down Barriers initiative across the Global Fund portfolio.	1	100	42
12.5. All countries are called on to invest in societal enabling approaches that remove legal barriers, shift harmful social and gender norms, reduce inequalities and improve institutional and community structures.	1	100	40
12.6. Countries should commit to removing and/or updating laws that impede HIV services and ensuring that no more than 10% of people living with HIV and people in key populations experience stigma and discrimination.	1	100	41
12.7. All countries are called on to learn about what works and does not work in the 20 countries supported by the Global Fund as part of its Breaking Down Barriers initiative and adopt country-owned strategic plans to reduce human rights-related barriers to services.	2	97	29
12.8. Co-action across development sectors is called for to support the three critical enablers of the HIV response—society, systems and services—to ensure that HIV services are non-discriminatory and person-centred. These are critical for stigma reduction and achieving national HIV goals.	2	100	30
12.9. National governments, research funders and development agencies are called on to adequately fund the development of evidence-based strategies to reduce HIV-related stigma and discrimination at scale.	2	100	30

* The N varies based on the number of respondents to a particular consensus point, as well as the number of respondents in each survey round. Fewer experts responded to the round 2 survey.

Given the high level of consensus in round 1, questions in round 2 focused on a further 31 questions that emerged from round 1 as potential consensus points for action. These comprised a combination of refined questions informed by the responses to round 1 questions and those with the next highest achievement of consensus (between 95% and 97%) in round 1.

### Consensus reached in survey round 2

Of the 31 points presented in round 2, 11 generated more than 95% consensus, ranging between 97% and 100% (table 1).

### Consensus statement

In the 2 survey rounds, the panel of experts reached consensus on 55 points within 12 themes (the theme commitment calls in addition to the 11 themes emerging from the literature review). The panel reached consensus on 11 points within the theme of funding of HIV-related stigma reduction at scale and on 9 commitment calls. Between 5 and 7 consensus points were reached on each of the following: HIV-related stigma measurement and assessment; stakeholder and community leadership; monitoring and evaluation (M&E); and knowledge gaps and research needs.

Between 2 and 4 consensus points were reached for each of the following 6 themes: the importance of addressing HIV-related stigma at scale; HIV-related stigma terms and definitions; frameworks to assess and guidance on implementation of HIV-related stigma and discrimination experienced in different settings; programming and approaches; community leadership in HIV-related stigma reduction implementation; and, intersectional stigma and discrimination. Consensus was reached on 1 point on M&E.

#### Theme 1: the importance of addressing HIV-related stigma at scale

The consensus statement endorses the importance of systematically measuring HIV-related stigma as a means of working towards and achieving targets. An important aspect of this is strengthening the understanding of how stigma and discrimination are currently experienced and expressed by different people in diverse settings, considering, for example, that in some languages, there are not different words to express each of these terms (as there are in English). The evidence base to inform stigma-reduction efforts must be up to date to reflect the dynamic and evolving nature of stigma.

#### Theme 2: HIV-related stigma terms and definitions

To improve learning across interventions and support efforts to assess progress towards global targets, the panel agreed that definitions and use of stigma-related language should be agreed on in the future through an inclusive consensus process.

#### Theme 3: frameworks

Although many different frameworks are used in different settings, consensus was reached on their value in research, implementation and policy development regarding HIV-related stigma and discrimination. Consensus was also achieved regarding frameworks based on underlying stigmatisation processes, and how they manifest, noting that such frameworks should be used more frequently in research, intervention development and policy on health-related stigmas.

#### Theme 4: programming and approaches

With regard to programming and approaches to reducing HIV-related stigma and discrimination at scale, the experts reached consensus on the importance of the efforts of the Global Partnership to bring together different stakeholders at country level to develop plans to tackle stigma, led by government and civil society, and the need for pre-service and in-service training of healthcare providers on the themes of HIV, human rights, key populations, stigma reduction, non-discrimination, gender sensitisation and ethics. Recognising the complex layers of intersectional identities, the panel also highlighted the fact that additional support is needed to strengthen skills and create spaces for diverse representatives from communities most affected by HIV-related stigma to meaningfully engage, influence, advocate and participate in decision-making for programme development in different countries.

#### Theme 5: community leadership in HIV-related stigma reduction implementation

The panel reached consensus on the point that the success of stigma-reduction efforts is limited when there is only restricted involvement of communities in all stages of investment decisions. Community leadership in providing education and counselling, facilitating access to HIV specialists and engaging support persons consistently contributes to the success of interventions to reduce HIV-related stigma.^26^ This combined approach should be scaled up in global stigma-reduction programming, and this consensus statement calls for a scaled-up, combined approach that reflects the evidence base to effectively address stigma reduction worldwide. To improve the sharing of lessons learnt and successful approaches, support is needed to ensure that community approaches are well documented and disseminated. Standard reporting guidelines should be developed on community engagement in HIV-related stigma reduction implementation.

#### Theme 6: intersectional and internalised stigma and discrimination

In response to intersectional stigma and discrimination, a recent systematic review found a range of sophisticated, intersectionality-informed interventions that were mostly successful at improving HIV, sexual health and empowerment-based outcomes, but less successful at reducing the aspects of stigma measured.^27^ Most studies assessed HIV-related (eg, ART adherence) and/or empowerment-based outcomes (eg, self-esteem and coping). The review found that although most interventions were multilevel and multistrategy, only five included a structural component.

Research has proven that a range of interventions can respond to internalised stigma, including the use of ART, social empowerment, economic strengthening and cognitive behavioural therapy; these result in consistent self-stigma reduction in low-income and middle-income countries.^19^

This consensus statement endorses interventions endeavouring to target a combination of structural-level and individual-level risks and resilience to tackle internalised stigma. Additionally, it highlights the fact that efforts are needed to legally protect people who belong to more than one marginalised group. It also emphasises that protective laws should be actively enforced and that reporting systems must be available to record abuse and seek redress. Importantly, synergistic attention should be paid to the areas of internalised stigma and stigma and discrimination within healthcare settings.

#### Theme 7: stigma and discrimination measures and assessment scales

Measures and assessment scales feature in seven of the points of the consensus statement. The panel reached consensus on the importance of researchers and implementers using community-led measures to monitor and evaluate stigma and discrimination reduction efforts, as well as on adapting existing standardised stigma instruments and measures to specific cultural contexts, for which guidance on local adaptation is also needed.

The People Living with HIV Stigma Index 2.0 (Stigma Index 2.0), designed by the community of people living with HIV for the community, measures multiple types of HIV-related stigma and its data are used by communities for advocacy purposes. The Stigma Index 2.0 has been used in many more countries and languages than were identified through a recent systematic review.^6^ This reflects that it may be commonly implemented, but that little has been reported in peer-reviewed publications. Consensus was reached that the results from the Stigma Index 2.0 should be included more often in articles and publications measuring different types of stigma and efforts supported that enable local partners to publish their findings.

#### Theme 8: monitoring and evaluation

Many of the countries where the expert panellists worked do not have robust M&E approaches to record HIV-related stigma and discrimination. Consensus was reached on supporting countries to develop M&E approaches to capture their situations.

#### Theme 9: stakeholder and community participation in M&E

Regarding stakeholder and community participation in M&E, a recent systematic review^26^ found that the geographical distribution of interventions varies by the type of stigma and discrimination being addressed. The experts reached consensus on the importance of investment to establish a robust evidence base across a range of settings and diverse populations and of promising interventions and processes to support stigma reduction. Consensus was reached on the need for more diverse approaches in evaluating the effectiveness of efforts to reduce stigma and discrimination.

Three consensus points highlighted the important role of community leadership: that community-led efforts to monitor stigma, discrimination and rights violations should be strengthened; that experiences of stigma should be monitored by community-led organisations; and that efforts must be scaled up to enable communities, among a range of M&E implementing partners, to monitor experiences of stigma, advocate for change as needed, and engage in and lead programme and policy development.

#### Theme 10: knowledge gaps and research needs

Consensus was reached on ten points regarding knowledge gaps and research needs, indicating that critical gaps in the literature must be addressed.

A 2023 systematic review and quantitative and qualitative comparative analysis of stigma interventions found that the geographic distribution of interventions varied by the type of stigma and discrimination being addressed.^7^ For internalised stigma and stigma and discrimination in laws and policies, around a quarter of studies were carried out in North America and the rest in low-income and middle-income countries. For stigma and discrimination in healthcare settings, studies were almost exclusively set in low-income and middle-income countries. This consensus statement highlights the need for investment to establish a robust evidence base across a range of settings and diverse populations and of promising interventions and processes to support stigma reduction.

The most common approaches to HIV-related stigma reported in the 2023 systematic review are education and counselling.^7^ Only a small number of studies included an awareness campaign or a total facility approach.^7^ Consensus was reached on the need to develop and evaluate more diverse approaches.

Consensus was also reached that more research is needed to inform the knowledge gap on the measurement and evaluation of the effectiveness of interventions to address internalised stigma.^19^

Until recently, there has been a lack of research on intersectional stigma.^27^ Recent focus on intersectional stigma has brought due attention to this analytical tool to explore and explain specific, qualitative differences that exist within already marginalised communities of people living with HIV.^28^ The expert panel agreed that more community-led research is needed about, by and for diverse communities of people living with and affected by HIV, including young people in low-income and middle-income countries.

Furthermore, the evidence base on addressing stigma and discrimination in laws and policies is particularly weak.^6^ In general, protective laws are found to be empowering, and laws that criminalise HIV exposure or behaviours relevant to HIV risk are detrimental. However, the importance of knowledge and implementation of the law in both cases was insufficiently highlighted: laws have an impact only insofar as they are implemented and enforced and as people know about them. Consensus was reached on the importance of stepping up efforts to strengthen the evidence and knowledge base about stigma and discrimination in laws and policies, especially understanding and responding to the extent to which laws are understood in different settings.

The evidence base on addressing internalised stigma and stigma and discrimination in healthcare has focused mainly on assessing individual-level outcomes, either among people living with HIV or among health facility staff (eg, types of stigma experienced and attitudes about people living with HIV and key populations) as the primary outcomes.^29^ In some cases, HIV-related biomedical outcomes (eg, viral suppression and ART adherence) are also included as outcomes.^29^ The consensus points highlighted the fact that stigma reduction efforts globally would be better served by including a broader range of outcomes, such as well-being, mental health, quality of life and flourishing, which supports previous recommendations in the literature.^9 29^

#### Theme 11: funding to address HIV-related stigma

While costing and cost-effectiveness research exists for many HIV interventions and social and behaviour change programmes, there is a dearth of evidence that specifically examines the cost effectiveness of approaches that address societal enablers for HIV outcomes.^29^ The panel reached agreement on the importance of conducting research to provide costing data to support decision-making on future investments across the large number of societal-enabling approaches that have been piloted and found to positively influence the effectiveness of HIV services.

The large number of consensus points reached on HIV-related stigma funding reflects the high level of concern globally about resourcing. Consensus was reached that in the future, organisations should track and report their stigma and discrimination-reduction investments. Agreement was reached on the importance of separately tracking investments to address HIV-related stigma and discrimination within broader HIV investments.

Overall, it was agreed that it is important for major funders to:

- Obtain guidance to incorporate due attention to stigma within investments and specific grant allocations.
- Ensure that diverse and inclusive community engagement is considered to guide investment priorities and/or dissemination of results from investments.
- Advocate for and convene other funders to enhance investment in stigma-reduction and discrimination-reduction efforts.

Efforts are needed in the future to explore opportunities for long-term stigma-reduction and discrimination-reduction investments and provide flexible funding mechanisms that could respond to emerging critical issues. The expert panel agreed on the recommendation that the global funding landscape of HIV-related stigma be regularly reviewed and coordinated nationally and that efforts are needed to integrate stigma and discrimination reduction within investments to strengthen tracking and reporting of these themes.

#### Theme 12: commitment calls

Of the nine commitment calls on which consensus was achieved, there was a strong focus on the need for countries to pledge to address the social and legal impediments to service access. Within this process, the consensus is to call for countries to commit to specific stigma-reduction and discrimination-reduction goals.

Societal and legal barriers hinder quality HIV prevention, care and treatment and support services, and must be removed. This, together with strong political and financial commitment, is essential for countries to reach their targets, and this consensus statement calls for this.

Without addressing stigma and discrimination, we will miss the UNAIDS 2025 targets by 2030, achieving only 91-88-93.^3^ The panel reached consensus to call for addressing HIV-related stigma and removing the societal and legal impediments to HIV services that are critical to achieving HIV targets and ending the AIDS pandemic as a public health threat by 2030.

The expert panel reached consensus on the importance of national governments, research funders and development agencies adequately funding the development of evidence-based strategies to reduce HIV-related stigma and discrimination at scale. Consensus points included calling on all countries to invest in societal-enabling approaches that remove legal barriers, shift harmful social and gender norms, reduce inequalities and improve institutional and community structures. Equally, the panel agreed that countries should commit to removing or updating laws that impede HIV services and ensuring that no more than 10% of people living with HIV and people in key populations experience stigma and discrimination.

The expert panel reached consensus on calling on countries to commit to specific goals, such as removing legal environments that impede HIV services and ensuring that no more than 10% of people living with HIV and people in key populations experience stigma and discrimination. It also reached consensus on calling for high-level commitment to scale up and improve the quality of programmes to reduce human rights-related barriers to HIV services by mainstreaming lessons learnt from the Breaking Down Barriers^30^ initiative across the Global Fund portfolio.

Consensus was reached on calling for co-action across development sectors, which is essential for ensuring the success of these critical enablers of the HIV response (ie, society, system and service enablers) in ensuring that HIV services are non-discriminatory and person centred. These are crucial for stigma reduction and achieving national HIV goals.^29^

### Call to action

The following call to action outlines the commitments that the expert panel urges for a variety of actors, including researchers, communities, funders, policy-makers and the trainers of healthcare professionals (box 1). It is based on a grouping of already published statements in the literature, which the expert panel prioritised in the consensus building process. Given the objectives of this study, no specific conclusions are drawn regarding the other statements from the evidence base (set out in the questions in online supplemental annex 4) that did not garner strong consensus.

Box 1 **Call to action to reduce HIV-related stigma at scale**

**For researchers:**

1. Systematically measure stigma to help meet global HIV targets.
2. Use validated measures for monitoring and evaluation of efforts to reduce stigma and discrimination.
3. Develop and evaluate diverse approaches to respond to evolving contexts and learning across disciplines on stigma and new and emerging themes and terminology.
4. Adapt existing standardised stigma instruments/measures to specific cultural contexts, for example, translating into appropriate terminology and local languages.
5. Incorporate research frameworks based on underlying stigmatisation processes and how they manifest.
6. Strengthen the evidence base on structural stigma, such as the role of law and policy, especially how laws are understood and interpreted in different settings.
7. Strengthen research on the measurement and evaluation of the effectiveness of interventions to address internalised stigma.
8. Conduct research to provide costing data to support future funding investment in the large number of societal enabling approaches that have been piloted and found to positively influence the effectiveness of HIV services.
9. Enable more community-led and authored research in low-income and middle-income countries, including supporting community-led organisations implementing the Stigma Index to disseminate their findings in the peer-reviewed literature (eg, through training about how the data can be analysed and interpreted in different settings).

**For health policy planners and medical schools:**

1. Train medical students and healthcare providers on HIV, human rights, inclusion and diversity, stigma reduction, non-discrimination, gender sensitisation and ethics in all preservice and in-service professional education.
2. Develop and disseminate guidance on local adaptation of measures.
3. Develop and disseminate standard reporting guidelines on community engagement.

**For implementers:**

1. Ensure that there are spaces that enable diverse community leadership, taking intersectionality into consideration, ensuring that communities can meaningfully engage, influence, advocate and participate in decision making for programme development in different countries.
2. Combine community-led approaches of providing education, counselling, facilitating access to HIV specialist advice and engaging a support person to scale up stigma-reduction programming.
3. Interventions should endeavour to target a combination of structural-level and individual-level risks and resilience to tackle internalised stigma.
4. Diversify approaches for tackling stigma and discrimination beyond education and counselling.
5. Track investments in HIV-related stigma and discrimination within broader HIV investments.
6. Offer support to networks implementing the Stigma Index 2.0 to disseminate their findings in the peer-reviewed literature.

**For funders:**

1. Support countries to develop robust monitoring and evaluation approaches to capture stigma and discrimination.
2. Ensure that diverse and inclusive community engagement is considered to guide investment priorities and/or dissemination of results from investments.
3. Invest in establishing a robust evidence base across a range of settings and diverse populations of promising interventions and processes to support stigma reduction.
4. Resource communities to monitor experiences of stigma, advocate for change as needed, and engage and lead in programme and policy development.
5. Regularly review the global funding landscape situation in relation to stigma.
6. Review and coordinate national funding landscapes in relation to stigma.
7. Obtain guidance to incorporate due attention to stigma within investments and specific grant allocations.
8. Track and report funders’ stigma-reduction investments.
9. Integrate stigma and discrimination reduction within investments and strengthen tracking and reporting.
10. Separately track investments in HIV-related stigma and discrimination within broader HIV investments.
11. Strengthen the global funding landscape to meet societal enabler targets.
12. Advocate for and convene other funders to enhance investment in stigma-reduction and discrimination-reduction efforts.
13. Invest in longer-term strategies to address stigma and discrimination and evaluate progress over time.
14. Provide flexible funding mechanisms to respond to emerging critical issues, evolving contexts and learning across disciplines.

**For policy-makers and duty bearers:**

1. Lead and support actions to reduce stigma and discrimination, including through the Global Partnership to eliminate HIV-related stigma.
2. Do more to protect people who belong to more than one marginalised group from violence, to report abuse and seek redress.
3. Develop a robust monitoring and evaluation approach to capture stigma and discrimination.
4. Enable communities to monitor experiences of stigma, advocate for change as needed, and engage and lead in programme and policy development.

**Specifically, we call on all countries and their cross-sectoral leadership to:**

1. Make concerted efforts to achieve UNAIDS 95-95-95 targets for HIV testing, treatment and viral suppression by 2030 and commit to addressing HIV-related stigma and removing societal and legal impediments to HIV services, in addition to scaling up treatment and prevention and improving social conditions.
2. Commit to specific goals, such as removing legal environments that impede HIV services and ensuring that no more than 10% of people living with HIV and people in key populations experience stigma and discrimination.
3. Invest in societal enabling approaches that remove legal barriers, shift harmful social and gender norms, reduce inequalities and improve institutional and community structures.
4. Remove and/or update laws that fuel stigma and impede HIV services.

The call to action has been developed to be of use to key actors to enable them to take responsibility and action of key priority areas. In the current geopolitical context affecting global health, accountability and effective action to address and reduce stigma is critical.

## Discussion

The large number of consensus points reached on HIV-related stigma funding reflects the high level of concern globally about resourcing. Given that the global funding landscape is insufficient to meet societal enabler targets,^24^ it is important to rectify this situation.

The majority of organisations (almost two-thirds) interviewed in a funding assessment commissioned by the IAS in 2021–2022 could not quantify their financial investments in HIV-related stigma and discrimination between 2017 and 2020. In addition, many funders with existing data on financial investments in HIV-related stigma and discrimination noted that their broader HIV investments included a component focused on mitigating stigma and discrimination, but these were typically not tracked separately (unpublished). Even in the latest National AIDS Spending Assessments country reports, the breakdown of spending towards social enablers is often not disaggregated to specific spending on reducing stigma and discrimination.^31^ As outlined above, consensus was reached that in future, organisations should track and report their stigma-reduction investments, including separately tracking investments in HIV-related stigma and discrimination within broader HIV investments.

As part of its Breaking Down Barriers initiative,^30^ the Global Fund provided intensive support throughout the duration of its 2017–2022 strategy to 20 countries to scale up programmes to remove barriers to health services. This was expanded to 24 countries in 2023–2024.^32^ All countries are called on to examine the lessons learnt from these 20 countries and similarly develop country-owned strategic plans to reduce human rights-related barriers to HIV services. In addition, all countries are called on to honour the commitments made at the 51st meeting of the UNAIDS Programme Coordinating Board meeting in 2022 for countries to join the Global Partnership for Action to Eliminate all Forms of HIV-Related Stigma and Discrimination.

A re-examination of Schwartländer *et al*’s 2011 HIV Investment framework^33^ by Stangl *et al*^30^ highlighted the importance of three critical enablers to addressing HIV—society, systems and services, referred to as the ‘3 Ss’. The experts reached consensus on calling for co-action across development sectors so that these critical enablers of the HIV response result in HIV services that are non-discriminatory and person-centred. These are essential for stigma reduction and achieving national HIV goals and are aligned with the findings from this consensus-building process.^30^

It is important to note that stigma is a process in which all actors, from individuals to institutions, have agency. They include those stigmatising, who have the opportunity to transform their values, attitudes and behaviours to be more inclusive and less judgemental. They include people experiencing stigma, who have the opportunity to seek and receive support and develop resilience and mechanisms for confronting and challenging stigma. And they include people who witness stigma in healthcare settings or in society, who can develop language and tools for being an ‘upstander’^34^ (as opposed to a more passive bystander) and calling out and confronting stigma when they see it. Rights holders and duty bearers can be the same people, just as those stigmatising and the stigmatised can be the same people. Everyone has the opportunity to contribute to reducing stigma and discrimination and mitigating their harmful effects.

This study brought together key global experts to review the evidence and seek consensus on priorities for action needed to address HIV-related stigma and discrimination at scale. Our adaptation deviated from traditional Delphi methodology in that the first round was designed to obtain consensus on priorities from existing statements and evidence from the literature on concepts, measures and approaches to HIV-related stigma, while the second round was designed to build consensus on prioritising action that the panel considered to be necessary.

While the engagement of an expert panel with diverse geographical, sociocultural and professional scope brought together the strengths of a wide range of experience, there were some limitations. While sharing a broad range of expertise in HIV and related stigma and discrimination, members of the panel had very different types of experience, ranging from academic research to policy and planning, clinical practice, project implementation, law, civil society, advocacy and activism. Interpretation of the English questionnaire may have been experienced differently according to native language, profession and experience. Given the extent of the evidence base, the first survey comprised more than 100 points of evidence for prioritisation. This may have limited participation, which decreased with the second survey. As agreement was very high on many of the first 100 survey questions, the consensus level was set after the results emerged, which is often not the case in Delphi studies and could be interpreted as the researchers controlling what should and should not be included.

Online surveys offer an efficient means of including a broad geographical range of expertise and opinions. However, computer access, literacy and electricity supply presented challenges for some panel members in resource-limited settings. An advantage of using a completely anonymous online software was that we were able to fully assure panellists that their responses were confidential. The disadvantage of complete anonymity was that we were unable to identify non-participants, which might have enabled us to more individually encourage and strengthen the response rate, particularly in the second survey round.

Despite the methodological limitations, consensus was extremely high overall from the earliest stages of the process. While 53 points within a consensus statement may be considered to be high, the outcome of the process provides rich guidance for policy and funding allocations needed to reduce HIV-related stigma at scale.

The timing of the publication of this article and its call to action coincides with political change in the USA and globally that has impacted negatively on the HIV funding landscape. While this may limit the ability of key actors to use the study outcomes and the call to action, as many jobs and research grants have been cut, it does not diminish the enduring importance of attention and action to reduce stigma.

## Conclusions

We recommend strong political and financial commitment to remove the societal and legal impediments that inhibit quality HIV prevention, care and treatment and support services; this is essential for countries to reach societal-enabler global targets. National governments, research funders and development agencies must adequately fund the development and implementation of evidence-based strategies to reduce HIV-related stigma and discrimination at scale.

The global funding landscape is insufficient to meet the 10-10-10 societal enabler targets, including reducing experiences of stigma and discrimination to less than 10% of people living with HIV and people in key populations. Strong political and financial commitments are essential.

This consensus statement demonstrates a need to establish a robust evidence base across a range of settings and diverse populations of promising interventions, partnerships and processes to support community-level, individual-level, organisational-level and public policy-level interventions. It also highlights the need to invest in societal-enabling approaches that remove legal barriers, shift harmful social and gender norms, reduce inequalities and improve institutional and community structures. As stated in the recommendations emerging from the findings of the consensus-building process, success of stigma-reduction efforts is possible only with the involvement of communities at all stages.

Action across sectors is needed to support the three critical enablers of the HIV response—society, systems and services—to ensure that HIV services are non-discriminatory and person-centred. These are essential for stigma reduction and achieving national HIV goals.

## Supplementary material

10.1136/bmjopen-2024-092516 online supplemental file 1

## Data Availability

All data relevant to the study are included in the article or uploaded as supplementary information.
