# Supplementary material for: Consensus on addressing HIV-related stigma and achieving the societal enabler targets using an adapted Delphi process
Source: BMJ Open. 2025 Aug 12;15(8):e092516. doi: 10.1136/bmjopen-2024-092516 (PMC12352172; doi:10.1136/bmjopen-2024-092516)
Supplement: online supplemental file 1 [file bmjopen-15-8-s001.docx]

**ANNEX 1. COUNTRY, AFFILIATION AND TITLE OF STEERING GROUP MEMBERS**

| **Title and affiliation** | **Country** |
| --- | --- |
| 1. Head of Programmes, Global Network of People Living with HIV (GNP+) | South Africa |
| 1. Co-Executive Director, Global Network of People Living with HIV (GNP+) | South Africa |
| 1. Adviser, UNAIDS | Egypt |
| 1. Director, HIV Programmes and Advocacy, IAS – the International AIDS Society Director, Watipa | Switzerland Australia |
| 1. Sr. Advisor for the Elimination of Stigma and Discrimination, Division of Global HIV and TB, Centers for Disease Control and Prevention Associate Faculty, Department of International Health, Johns Hopkins Bloomberg School of Public Health | United States |

**ANNEX 2. COUNTRY, AFFILIATION AND TITLE OF EXPERT PANEL MEMBERS**

| **Title and affiliation** | **Country** |
| --- | --- |
| 1. Representative, Y+, Kenya | Kenya |
| 1. Director, Office of Global Health, Drexel University | United States |
| 1. Associate Professor, Pediatrics, Johns Hopkins University | United States |
| 1. Social Behavioural Division Lead; Desmond Tutu Health Foundation, University of Cape Town | South Africa |
| 1. Professor, Epidemiology, Johns Hopkins University | United States |
| 1. Executive Director, HIV Justice Network | Netherlands |
| 1. Senior Behavioral Scientist, Rand Corporation | United States |
| 1. ICW Global, International Community of Women Living with HIV | United States |
| 1. Associate Professor, University of California | United States |
| 1. Managing Director, Asia Pacific Council of AIDS Service Organizations, | Malaysia |
| 1. Deputy Board Chair, Y+ Global | United Kingdom |
| 1. Adjunct Associate Professor, Columbia University | United States |
| 1. Policy Advisor, Gender Equality, Health and HIV, UN Women | United States |
| 1. Visiting Research Fellow, Coventry University | United Kingdom |
| 1. Gender Equality Officer, La Comunidad Internacional de Mujeres Viviendo con VIH/SIDA | Argentina |
| 1. Associate Professor, Human Development and Family Sciences, University of Delaware | United States |
| 1. Policy Specialist: Law, Human rights and gender, UNDP | United States |
| 1. Regional Coordinator, Stigma and Discrimination, West and Central Africa, UNAIDS | Nigeria |
| 1. Alternate member, Global Fund, Developing Country NGO Delegation | Jamaica |
| 1. Latin American and Caribbean representative, IAS Governing Council | Brazil |
| 1. Director, Institute on Inequalities in Global Health, University of Southern California | United States |
| 1. Head of Programmes, Réseau National des Associations de PVVIH du Sénégal | Senegal |
| 1. ARASA, Deputy Director | Namibia |
| 1. Board member, OutRight Action International | United States |
| 1. Technical Advisor, Global Fund to Fight AIDS, Tuberculosis and Malaria | Switzerland |
| 1. Africa NGO delegate, UNAIDS Africa NGO Group | Nigeria |
| 1. Professor, Medicine, University of Malaya | Malaysia |
| 1. Associate Faculty Director, Harvard Global Health Institute | United States |
| 1. Chairperson, Africa Network for People Who Use Drugs | Tanzania |
| 1. Policy Advisor, Gender Equality, Health and HIV, UN Women | United States |
| 1. Research Chair in Global Health Equity and Social Justice with Marginalized Populations, University of Toronto | Canada |
| 1. Manager, SDGs and UNAIDS, HIV, Health and Development Group, UNDP | United States |
| 1. Research Director and Principal Investigator, Centre for Sexual Health and HIV/AIDS Research | Zimbabwe |
| 1. Project Director, PSI | South Africa |
| 1. Director, Programmes and Technical (Positive Action), ViiV Healthcare | United Kingdom |
| 1. Young Leader 2021, International AIDS Society | Kenya |
| 1. Executive Director and Founding Member, International Community of Women Living with HIV Eastern Africa | Uganda |
| 1. Co-founder, Gestos | Brazil |
| 1. Global Advocacy Manager, Global Network of People Living with HIV | South Africa |
| 1. Senior Technical Advisor, Global Health Division, RTI International | United States |
| 1. Coordinator, Africa Key Populations Expert Group | Kenya |
| 1. Treasurer, ICW Global International Steering Committee, International Community of Women Living with HIV | Uganda |
| 1. Executive Director, Institute of HIV Research and Innovation | Thailand |
| 1. Adjunct Assistant Professor, Columbia University | United States |
| 1. ICW AP Regional Coordinator, International Community of Women Living with HIV | Nepal |
| 1. Chief Executive Officer, Human Sciences Research Council | South Africa |
| 1. Associate Professor, Department of Public Health, University of Copenhagen | Denmark |
| 1. Chair, United States People Living with HIV Caucus | United States |
| 1. Chief, Community Mobilization, UNAIDS | Switzerland |
| 1. Founder, Transgender Women of Africa | South Africa |

**ANNEX 3: LITERATURE SOURCES INFORMING CONSENSUS POINTS**

| 1. Ferguson L, Gruskin S, Bolshakova M, Yagyu S, Fu N, Cabrera N, Rozelle M, Kasoka K, Oraro-Lawrence T, Stackpool-Moore L, Motala A, Hempel S. Frameworks and measures for HIV-related internalized stigma, stigma and discrimination in healthcare and in laws and policies: a systematic review. J Int AIDS Soc. 2022 Jul;25 Suppl 1(Suppl 1):e25915. doi: 10.1002/jia2.25915. PMID: 35818866; PMCID: PMC9274352 [6] 2. Hempel S, Ferguson L, Bolshakova M, Yagyu S, Fu N, Motala A, Gruskin S. Frameworks, measures, and interventions for HIV-related internalised stigma and stigma in healthcare and laws and policies: systematic review protocol. BMJ Open. 2022 Dec 9;11(12):e053608. doi: 10.1136/bmjopen-2020-053608 [12] 3. Joint United Nations Programme on HIV/AIDS. Addendum to: Evidence for eliminating HIV-related stigma and discrimination — Guidance for countries to implement effective programmes to eliminate HIV-related stigma and discrimination in six settings - Addressing intersectional stigma and discrimination in national HIV responses. Geneva: UNAIDS; 2022. [13] 4. Joint United Nations Programme on HIV/AIDS. Evidence for eliminating HIV-related stigma and discrimination - Guidance for countries to implement effective programmes to eliminate HIV-related stigma and discrimination in six settings. Geneva: UNAIDS; 2020. Available from: <https://www.unaids.org/en/resources/documents/2020/eliminating-discrimination-guidance> [14] 5. Joint United Nations Programme on HIV/AIDS. Global Partnership for Action to Eliminate All Forms of HIV-related stigma and discrimination brochure. Geneva: UNAIDS; 2020. Available from: <https://www.unaids.org/sites/default/files/media_asset/global-partnership-hiv-stigma-discrimination_en.pdf> [15] 6. Joint United Nations Programme on HIV/AIDS. Global Partnership for Action to Eliminate all Forms of HIV-related Stigma and discrimination. Geneva: UNAIDS; 2018. Available from: <https://www.unaids.org/sites/default/files/media_asset/global-partnership-hiv-stigma-discrimination_en.pdf> [16] 7. Joint United Nations Programme on HIV/AIDS. Monitoring and evaluating programmes to eliminate HIV and key population stigma and discrimination in six settings: Guidance provided by the Global Partnership for Action to Eliminate all Forms of HIV-related Stigma and Discrimination. Geneva: UNAIDS; 2023. [17] 8. Mak WWS, Mo PKH, Ma GYK, Lam MYY. Meta-analysis and systematic review of studies on the effectiveness of HIV stigma reduction programs. 2017 Jul 1; Available from: <https://pubmed.ncbi.nlm.nih.gov/28704645/> [18] 9. Pantelic M, Steiners J, Park JJH, Mellors S, Murau F. 'Management of a spoiled identity': systematic review of interventions to address self-stigma among people living with and affected by HIV. BMJ Glob Health. 2018 Mar 1; Available from: <https://pubmed.ncbi.nlm.nih.gov/30997170/> [19] 10. Rao D, Elshafei A, Nguyen M, Hatzenbuehler ML, Frey S, Go VF. A systematic review of multi-level stigma interventions: state of the science and future directions. BMC Med. 2019 Feb 15;17(1):41. doi: 10.1186/s12916-018-1244-y [8] 11. Sengupta S, Banks B, Jonas D, Miles MS, Smith GC. HIV interventions to reduce HIV/AIDS stigma: a systematic review. AIDS Behav. 2011 Aug; Available from: <https://www.ncbi.nlm.nih.gov/pmc/articles/PMC3128169/pdf/nihms269079.pdf> [20] 12. Smythe T, Adelson JD, Polack S. Systematic review of interventions for reducing stigma experienced by children with disabilities and their families in low- and middle-income countries: state of the evidence. Trop Med Int Health. 2020 Mar 7; Available from: <https://onlinelibrary.wiley.com/doi/full/10.1111/tmi.13388> [21] 13. Stangl AL, Lloyd JK, Brady LM, Holland CE, Baral S. A systematic review of interventions to reduce HIV-related stigma and discrimination from 2002 to 2013: how far have we come? 2013 Nov 13; Available from: <https://www.ncbi.nlm.nih.gov/pmc/articles/PMC3833106/> [22] 14. Stangl AL, Singh D, Windle M, Sievwright K, Footer K, Iovita A, Mukasa S, Baral S. A systematic review of selected human rights programs to improve HIV-related outcomes from 2003 to 2015: what do we know? BMC Infect Dis. 2019 Mar 5;19:206. doi: 10.1186/s12879-019-3692-1. [23] 15. Stover J, Glaubius R, Teng Y, Kelly S, Brown T, Hallett TB, Revill P, Bärnighausen T, Phillips AN, Fontaine C, Frescura L, Izaola-Licea JA, Semini I, Godfrey-Faussett P, De Lay PR, Benzaken AS, Ghys PD. Modeling the epidemiological impact of the UNAIDS 2025 targets to end AIDS as a public health threat by 2030. PLoS Med. 2021 Oct 18;18(10):e1003831. doi: 10.1371/journal.pmed.1003831 [3] 16. The Global Fund. Questions and Answers: Breaking Down Barriers to Access: Scaling up Programs to Remove Human Rights-Related Barriers to Health Services in 20 Countries and Beyond. Geneva: The Global Fund; 2020 Jun 8. Available from: <https://www.theglobalfund.org/media/1213/crg_breakingdownbarriers_qa_en.pdf> [24] |
| --- |

**ANNEX 4. Full list of round one questions**

Sector

Please indicate which sector you represent?

*Civil society / Academia / Clinical practice / Law / Donor / Policy / Other (please state)*

The importance of addressing HIV-related stigma at scale

1. Stigma and discrimination are known barriers to access to HIV (and other) services for people living with HIV and key populations. Recent modelling has estimated that reaching the UNAIDS societal enabler targets (which include ‘less than 10% of people living with HIV and key populations experience stigma and discrimination’) will prevent 2.5 million new infections and 1.7 million AIDS-related deaths by 2030.

How important do you feel that it is to understand how stigma and discrimination are being experienced?
*Very important / Important / Moderately important / Slightly important/ Unimportant*

2. How important do you feel it is to measure stigma in a systematic and thorough manner in order to reach global HIV targets?
 *Very important / Important / Moderately important / Slightly important/ Unimportant*

3. To what extent do you agree or disagree that it is important to have rigorous measurement across different spheres and types of stigma (e.g. internalised, etc) in different settings?

*Strongly agree / Agree / Undecided / Disagree / Strongly disagree*

4. What do you think has been the main barrier hampering efforts to get to the heart of stigma at scale?
 *Open comment*

HIV related stigma terms and definitions

5. Analysis of stigma interventions to date show that some researchers and practitioners use different definitions of HIV-related stigma. There is also a general lack of consensus on key aspects of what stigma actually is and consequently how to address it. This makes it difficult to learn across interventions.

Do you feel it is important to achieve consensus on definitions and use of stigma-related language?

*Very important / Important / Moderately important / Slightly important / Unimportant*

6. How important do you feel that achieving consensus on definitions would be in enabling comparability, cross-setting learning and efforts to assess progress towards global targets?

*Very important / Important / Moderately important / Slightly important / Unimportant*

7. Do you think there is an urgent need for a universally agreed-upon list of types of HIV-related stigma and definitions?

*Yes / No / No opinion (+ open comment)*

8. Is there anything else that you feel is important in relation to HIV-related stigma terms and definitions?

*Open comment*

Frameworks to assess and guidance on implementation of HIV-related stigma and discrimination experienced in different settings

9. Conceptual frameworks are frequently used as a tool to assess stigma in different settings.

To what extent do you consider frameworks to be useful in research, real-world intervention development, and policy on health-related stigmas?

*Very useful / Useful / Moderately useful / Not very useful / Not useful at all*

10. It is widely acknowledged that stigma manifests in different ways.

To what extent do you think it is necessary to use conceptual frameworks that target specific types of stigma (as compared to more general frameworks that are adaptable to different contexts)?

*Highly necessary / Necessary / Neither necessary or unnecessary / Not necessary / Completely unnecessary*

11. Do you feel that it would be preferable for researchers to use general frameworks based on the underlying stigmatization process and how it manifests, rather than their own unique frameworks for each study?

*Yes / No / No opinion (+ open comment)*

12. The Health Stigma and Discrimination Framework (HSDF) provides an HIV framework that includes the health care setting, the legal policy setting and the individual (internalised and experienced stigma) as well as all of the socioecological levels. It has been included in UNAIDS and PEPFAR guidance.

Do you feel this should be used as a common framework in research, intervention development, and policy on health-related stigmas?

*Yes / No / No opinion (+ open comment)*

13. To what extent do you feel this framework should be frequently used as a common framework in research, intervention development, and policy on health-related stigmas?

*Always / Often / Sometimes / Rarely / Never*

14. Are there any other elements, not included, that you feel should be covered by the Health Stigma and Discrimination Framework?

*Yes / No / No opinion (+ open comment)*

15. Guidance provided by the Global Partnership for Action to Eliminate all Forms of HIV-related Stigma and Discrimination is organized according to six settings: community, workplace, education, health care, justice, and emergency*.

Do you think it is feasible to implement stigma reduction programming in all settings (*NB. “emergency” will depend upon the particular country context)?

*Yes / No / No opinion (+ open comment)*

16. Are there other settings, not mentioned here, that you think should be highlighted?

*Open comment*

Programming and approaches

17. Do you feel that it is important for all pre-service and in-service healthcare providers to be trained on HIV, human rights, key populations, stigma reduction, non-discrimination, gender-sensitization and ethics?

*Very important / Important / Moderately important / Slightly important / Unimportant*

18. Do you feel one of the topics (HIV, human rights, key populations, stigma reduction, non-discrimination, gender-sensitization and ethics) is more important than others to include in pre- and in-service education for healthcare providers, professionals, duty-bearers and community leaders?

*Open comment*

19. Would your answer be different if considering healthcare providers, professionals, duty-bearers or community leaders?

*Open comment*

20. Do you feel that support is needed to strengthen skills and create spaces for multiply-marginalized populations to meaningfully engage, influence, advocate and participate in decision-making for programme development in different countries?

*Yes / No / No opinion*

Community participation in HIV-related stigma reduction implementation

21. Community participation, in providing education, counselling, facilitating access to an HIV specialist, and engaging a support person in combination have been found to consistently contribute to the success of interventions to reduce HIV-related stigma.

How important do you feel it is to ensure that this combination of intervention is scaled-up in global stigma-reduction programming?

*Very important / Important / Moderately important / Slightly important / Unimportant*

22. Only half of studies from a global systematic review on stigma interventions report on community engagement in the intervention design or research process.

Do you feel that we should call for standard reporting guidelines on community engagement would enable transparency on the extent to which and how community engagement occurs in practice?

*Yes / No / No opinion*

23. There is some ambiguity in the research literature about the key elements of meaningful involvement of people living with HIV.

How important do you feel it is for this component to be well documented?

*Very important / Important / Moderately important / Slightly important / Unimportant*

24. Please list any examples you are familiar with of good practice guidelines for documenting and reporting meaningful community engagement.

*Open comment*

Intersectional stigma and discrimination

25. Very little research examines intersectional stigma in the context of HIV.

Can we adequately measure or assess intersectional stigma without a deep understanding of how it manifests in the context of HIV?

*Yes / Somewhat / No / No opinion*

26. In order to reduce stigma as scale, do you agree that synergistic attention is required in the areas of internalized stigma; stigma and discrimination within healthcare settings?

*Yes / No / No opinion*

27. Do you agree that to reduce stigma at scale, synergistic attention is required in the areas of internalized stigma; stigma and discrimination in policy?

*Yes / No / No opinion*

28. Do you agree that to reduce stigma at scale, synergistic attention is required of the areas of internalized stigma; stigma and discrimination in laws?

*Yes / No / No opinion*

29. Do you feel that intersectional stigma and discrimination are adequately addressed at the levels of research and policy?

*Yes / No / No opinion*

30. Do you feel intersectional stigma and discrimination are adequately taken into consideration in national programming?

*Yes / No / No opinion*

31. Do you feel that there are adequate intersectional stigma and discrimination reduction interventions that recognize and name how systems of power, privilege, and oppression impact individual experiences and fuel intersectional stigma and discrimination?

*Yes / No / No opinion (+ open comment)*

32. Do you feel that there are adequate intersectional stigma and discrimination reduction interventions that are designed to dismantle and mitigate the harm caused by those systems?

*Yes / No / No opinion (+ open comment)*

33. Do you feel that there are adequate intersectional stigma and discrimination reduction interventions that ensure community leadership and meaningful engagement?

*Yes / No / No opinion (+ open comment)*

34. Do you feel that there are adequate intersectional stigma and discrimination reduction interventions that support collective action, cohesion and resistance to address the intersecting axes of inequality experienced by multiply-marginalized populations?

*Yes / No / No opinion (+ open comment)*

35. Do you feel that our understanding of intersectional stigma has expanded since the Health Stigma and Discrimination Framework was published and that this now needs to be updated?

*Yes / No / No opinion*

36. The majority of multi-level stigma interventions studied have focused on intrapersonal and interpersonal levels. Do you agree that more research is needed to incorporate community-, organizational-, and structural level influences into multi-level stigma interventions?

*Yes / No / No opinion*

37. Do you agree that a stronger focus is needed on policy-level interventions to address stigma at the institutional and structural levels?

*Yes / No / No opinion*

38. Do you feel there is adequate support (e.g. capacity strengthening, training, etc.) to people that belong to more than one marginalized group with lived experience of intersectional stigma and discrimination to recognize, cope with, and share how intersectional stigma and discrimination influence their health and lives more broadly?

*Yes / No / No opinion*

39. Research shows that ART provision, social empowerment, economic strengthening and cognitive-behavioural therapy interventions result in consistent self-stigma reductions in low- and middle-income countries.

Do you feel that Interventions should endeavour to target a combination of structural-level and individual-level risks and resilience to tackle internalized stigma?

*Yes / No / No opinion*

40. Do you feel that more needs to be done to protect people that belong to more than one marginalized group from violence, but that such laws are enforced, and that reporting systems are available to report abuse and seek redress?

*Yes / No / No opinion*

41. Do you believe that the full reach and impact of HIV-related stigma reduction efforts remain limited by a nearly exclusive focus targeting only one level of analysis?

*Yes / No / No opinion*

Measures (e.g. assessment scales)

42. Although individual studies have shown different interventions to be effective in reducing stigma, the outcome measures lack uniformity and validity, making both interpretation and comparison of study results difficult.

Do you agree that there is a need to establish standard stigma reduction outcome measures?

*Yes / No / No opinion*

43. How important do you think it is for researchers and programmers to use validated measures for M&E of stigma and discrimination reduction efforts?

*Very important / Important / Moderately important / Slightly important / Unimportant*

44. How important do you think it is for researchers and programmers to use community-led measures for M&E of stigma and discrimination reduction efforts?

*Very important / Important / Moderately important / Slightly important / Unimportant*

45. How important do you feel it is to adapt existing standardized stigma instruments/measures to specific cultural contexts?

*Very important / Important / Moderately important / Slightly important / Unimportant*

46. Do you feel that adequately clear and comprehensive HIV-related stigma measures are available to you to confidently and regularly use in your work?

*Yes / No / No opinion*

47. Some measures address multiple types of stigma, including the Integrated Biological and Behavioral Surveys (IBBS) and the Stigma Index 2.0, designed by the community for the community. The Stigma Index 2.0 website states that it has been used in many more countries and languages than were found through the IAS literature review, suggesting that it is commonly used, but little has been written about it in peer-reviewed publications.

Do you feel that the Stigma Index needs to be better represented in publications?

*Yes / No / No opinion*

48. Do you think it is important that community organizations implementing the Stigma Index are supported to disseminate their findings in the peer-reviewed literature (e.g. through training about how the data can be analyzed/interpreted in different settings? Why?

*Very important / Important / Moderately important / Slightly important / Unimportant*

49. How helpful would it be to have guidance on local adaptation of measures?

*Very helpful / Helpful / Moderately helpful / Slightly helpful / Not helpful at all*

50. Only the National Commitments and Policy Instrument (NCPI) has been identified to focus on stigma and discrimination in healthcare and in law and policy. It contains a range of relevant indicators on experiences of stigma and discrimination in healthcare, laws that might be discriminatory and laws that protect against HIV-related discrimination.

Do you feel this is adequate?

*Yes / No / No opinion*

51. Overall, there is a lack of measures relating to HIV stigma and discrimination in law and policy and protective laws. This may be due to the complexity and sensitivity of measuring these topics as well as the extensive investment that would be required to do this effectively at scale. Data are increasingly available about the existence of discriminatory laws and policies, but little has been published about measuring and evaluate their implementation to identify if, when or how these processes and structures have impacts at the healthcare and personal levels.

Do you feel that additional effort is needed to measure and evaluate the implications of discriminatory laws and the mechanisms of their impacts at the individual and health systems levels?

*Yes / No / No opinion*

Monitoring and evaluation

52. The 2022 Global Partnership’s draft guidance states that having a robust monitoring and evaluation strategy in place is critical to ensure the rapid and appropriate allocation of resources to address stigma and discrimination.

Do you feel that all countries have this in place?

*Yes / To some extent / No / No opinion*

Stakeholder and community participation in M&E

53. In addition to other forms of measurement and data collection, do you feel that experiences of stigma should be monitored by community-led organisations?

*Yes / No / No opinion*

54. Do you feel that efforts need to be scaled-up to enable local networks of people living with HIV, key populations, indigenous populations, people in prisons and other people in detention, migrants, and women and girls, particularly adolescent girls and young women—to monitor experiences of stigma and to advocate for change as needed, and engage in programme and policy development?

*Yes / No / No opinion*

55. Is your answer similar or different on this question compared to all other aspects of the HIV response?

56. How important do you feel it is that community-led efforts be increased to monitor stigma, discrimination and rights violations experienced by people living with HIV, key populations, indigenous populations, people in prisons and other people in detention, migrants, and women and girls, particularly adolescent girls and young women?

*Very important / Important / Moderately important / Slightly important / Unimportant*

Knowledge gaps and research needs

57. Do you feel that critical challenges and gaps remain which are impeding the identification of effective stigma-reduction strategies that can be implemented by national governments on a larger scale?

*Yes / No / No opinion*

58. Do you feel that critical challenges and gaps remain which hamper the effectiveness of donor support to national stigma-reduction strategies?

*Yes / No / No opinion*

59. There is a lack of research on intersectional stigma, which occurs when different types of stigma are compounded due to belonging to more than one marginalised social group.

Do you feel that more implementation research is needed to guide how countries address this issue and support people with intersecting vulnerabilities?

*Yes / No / No opinion*

60. Do you feel that there is alignment about what intersectional stigma refers to? (+ open comments)

*Yes / No / No opinion*

61. Do you feel that the absence of studies examining internalized stigma related to TB, viral hepatitis or other co-infections often experienced people living with HIV needs to be addressed? (+ open comments)

*Yes / No / No opinion*

62. The vast majority of HIV-related stigma research has focused on general populations of people living with HIV or pregnant women living with HIV.

Do you agree that more research is needed on key populations and young people in low and middle income countries?

*Yes / No / No opinion*

63. Do you feel that more research is urgently needed to identify the most effective approaches among key populations affected by HIV and young people, as well as what approaches might help reduce intersectional internalized-stigma?

*Yes / No / No opinion*

64. A recent systematic review found that the geographic distribution of interventions varies by the type of stigma (and discrimination) being addressed. For internalized stigma and stigma and discrimination in law and policy, around a quarter of studies have been carried out in the USA, with the rest in low- and middle-income countries. For stigma and discrimination in healthcare settings, studies were almost exclusively set in low- and middle-income countries.

How important is it that investment is made to establish a robust evidence base across a range of settings and diverse populations, of promising interventions and processes to support stigma reduction?

*Very important / Important / Moderately important / Slightly important / Unimportant*

65. The most common approaches to HIV-related stigma reported in the academic literature are education and counselling. A very small number of studies included an awareness campaign or a total facility approach.

Do you feel that we should highlight the need for more diverse approaches – and accompanying evaluations of their effectiveness? (+ open comments)

*Yes / No / No opinion*

66. Educational programs alone are often ineffective in reducing stigmatizing attitudes in members of the public, and the little resulting stigma reduction that occurs may be short-lived and superficial.

Do you agree that future research on multi-level stigma interventions is needed to explore a wider range of stigma-reduction strategies and to utilize evidence-based strategies that prior research has shown to be effective in reducing stigma?

*Yes / No / No opinion*

67. Do you agree that future research is needed to evaluate how changes at one level of stigma (e.g., intrapersonal) impact other levels of stigma (e.g., community) to guide the development of more effective multi-level interventions, to identify mechanisms of change in multi-level stigma interventions, and to explore the barriers and facilitators to the dissemination of multi-level stigma interventions across diverse contexts?

*Yes / No / No opinion*

68. The evidence base around addressing stigma and discrimination in law and policy is particularly weak. In general, protective laws are found to be empowering and laws that criminalise HIV exposure or behaviours relevant to HIV risk detrimental, but the importance of knowledge and implementation of the law in both cases was insufficiently highlighted: laws only have an impact insofar as they are implemented and that people know about them.

Do you feel that efforts should be stepped-up to strengthen the evidence and knowledge base, especially in terms of the extent to which laws are understood in different settings?

*Yes / No / No opinion*

69. The evidence base on addressing internalized stigma and stigma and discrimination in healthcare, has focussed mainly on HIV-related clinical outcomes as the primary outcome.

Do you feel that stigma reduction efforts globally would be better served by including broader outcomes such as mental health, quality of life and flourishing?

*Yes / No / No opinion (+ open comment)*

70. Do you feel there is sufficient evidence about the various types of stigma experienced by different people in different contexts?

*Yes / No / No opinion*

71. A recent systematic review has found that more than a quarter of examined interventions were ineffective in reducing self-stigma.

Do you feel that specifically in relation to internalized stigma, it may be more challenging to measure progress and evaluate the effectiveness of interventions?

*Yes / No / No opinion*

72. We currently know very little about the extent to which and how changes in stigma reduction affect associated health outcomes, such as increasing HIV testing, increasing access to HIV treatment/ care, improving policy support for people living with HIV, improving mental health outcomes and social support, and/or reducing HIV-related symptoms.

How important do you feel that research to inform this gap is?

*Very important / Important / Moderately important / Slightly important / Unimportant*

73. While costing and cost-effectiveness research exists for HIV interventions and social and behaviour change programs, there is a dearth of evidence that specifically examines the cost-effectiveness of approaches that address societal enablers for HIV outcomes. Cost-effectiveness analysis compares the cost per unit outcome (e.g. new HIV diagnosis, new treatment initiation, new client virally suppressed, etc.) between two or more programmes.

How important do you feel need for research to provide such data to support future funding investment in the large number of societal enabling approaches that have been piloted and found to positively influence the effectiveness of HIV services?

*Very important / Important / Moderately important / Slightly important / Unimportant*

HIV-related stigma funding

74. A 2021 stigma funding landscape assessment found there to be vocal support for stigma reduction at the global and institutional level and consolidation of strategic focus on societal enabler targets among some stakeholder groups.

In terms of funding, it found focus areas with the highest levels of investment to be:

- Stigma reduction in healthcare settings
- Duty-bearer sensitization training
- Community-led advocacy
- Community-led stigma measurement

Focus areas with lower levels of investment were identified as:

- Key population stigma reduction
- Empowerment of adolescent girls and young women
- Violence reduction
- Internalized stigma reduction

Under-represented areas in the current funding landscape were identified as:

- Structural interventions to reduce stigma, such as addressing criminalization
- Harm-reduction for people who inject drugs
- Intersectional stigma, including key population stigma

Do these findings resonate with your perspective of the current funding landscape?

*Yes / No / No opinion (+ open comment)*

75. Do you agree that it would be useful to regularly review the global funding landscape situation in relation to stigma?

*Strongly agree / Agree / Undecided / Disagree / Strongly disagree*

76. Do you agree that it would be useful to review and coordinate national funding landscapes in relation to stigma?

*Strongly agree / Agree / Undecided / Disagree / Strongly disagree*

77. The majority of organizations interviewed in a recent funding assessment (almost two thirds) could not quantify their financial investment in HIV-related stigma, and discrimination, in 2017-2020.

Do you feel that organization should track and report their stigma-reduction investments?

*Yes / No / No opinion*

78. How important do you feel this is?

*Very important / Important / Moderately important / Slightly important / Unimportant*

79. Many of the funders with existing data on financial investment in HIV-related stigma, and discrimination noted that their broader HIV investments included a component focused on mitigating stigma, and discrimination, but that these were typically not tracked separately.

How important do you think it is to track separately investments in HIV-related stigma, and discrimination within broader HIV investments? Why?

*Very important / Important / Moderately important / Slightly important / Unimportant (+ open comments)*

80. Do you agree that all investments in research and programmes in relation to HIV should include a component to address stigma; and to measure any influence of stigma on the results?

*Yes / No / No opinion (+ open comments)*

81. The global funding landscape is insufficient to meet societal enabler targets.

How important do you feel it is to rectify this situation?

*Very important / Important / Moderately important / Slightly important / Unimportant*

82. What mechanisms would you suggest to increase funds to meet the societal enabler targets?

83. Do you agree that the limited involvement of communities in all stages of investment decisions threatens limits the success of stigma reduction efforts?

*Yes / No / No opinion*

84. Do you feel it would be helpful to develop a “united voice” amongst funders of HIV-related stigma reduction?

*Yes / No / No opinion*

85. Do you feel that a major donor should lead this?

*Yes / No / No opinion*

86. Do you feel that any of the following would be important among major donors:

- - Mainstream and/or scale up attention to stigma within all investments
  - Set a common commitment and quantify allocation within all investments (>10%)
  - Obtain guidance to incorporate due attention to stigma within investments and specific grant allocations
  - Ensure diverse and inclusive community engagement is considered to guide investment priorities and/or dissemination of results from investments
  - Advocate for and convene other funders to enhance investment in stigma, and discrimination, reduction effort

*Yes / No / No opinion*

87. Do you feel that major donors are needed to:

- - Support existing global processes and partnerships to reduce HIV-related stigma?
  - Co-fund existing coordinating frameworks, e.g. UNAIDS societal enabler targets or the Global Partnership for Action to Eliminate All Forms of Stigma and Discrimination related to HIV?

*Yes / No / No opinion*

88. Do you feel that efforts are needed to:

- - Consolidate investments with other sectors focusing on stigma, and discrimination?
  - Co-fund national government investment in stigma?
  - Explore opportunities for long-term stigma, and discrimination reduction investments?
  - Integrate stigma, and discrimination, within investments and strengthen tracking and reporting?

*Yes / No / No opinion*

89. Do you feel that opportunities should be leveraged to:

- - Invest in current research gaps in order to catalyse investment in stigma reduction at scale?
  - Provide flexible funding mechanisms that could respond to emerging critical issues?
  - Leverage intermediary grant makers to reach local organizations involved in the stigma response?

*Yes / No / No opinion*

Commitment calls

90. Mathematical modelling indicates that the achievement of the UNAIDS targets of 95–95–95 HIV testing, treatment and viral suppression rates by 2030, might only be possible to achieve to the extent of 91– 88–93 without addressing stigma. Achieving these targets will require concerted efforts to scale up treatment for all people living with HIV and effective prevention measures for populations who most need them and to improve social conditions to remove barriers to progress. These targets are ambitious but not impossible. They have already been achieved in some countries and in some populations.

Do you agree that addressing stigma and removing the societal and legal impediments to HIV services are critical to achieving HIV targets and ending the AIDS epidemic as a public health threat by 2030?

*Strongly agree / Agree / Undecided / Disagree / Strongly disagree*

91. Societal and legal impediments inhibit quality HIV prevention, care, and treatment and support services and need to be removed. The political declaration adopted by UN member countries at the high-level meeting on HIV and AIDS in June 2021, included new societal enabler global targets for achievement by 2025 that will address this gap.

Do you feel that strong political and financial commitment is essential for countries to reach these local targets?

*Yes / No / No opinion*

92. Do you feel that countries should commit to specific goals such as removing legal environments that impede HIV services; and ensure that no more than 10% of people living with HIV and key populations experience stigma and discrimination?

*Yes / No / No opinion*

93. As part of its Breaking Down Barriers initiative, the Global Fund is providing intensive support throughout the duration of the 2017-2022 Strategy to 20 countries where needs, opportunities, capacities and partnerships provide real possibilities for scale-up that will result in important gains for the health of those affected. As of June 2020, eight of the 20 countries in the initiative had adopted country-owned, strategic plans to reduce human rights-related barriers to services, with many others expected to adopt such plans.

Do you feel that all countries should learn about what works and does not work in these 20 countries and adopt country-owned, strategic plans to reduce human rights-related barriers to services?

*Yes / No / No opinion*

94. How important do you feel it is to scale-up and improve the quality of programs to reduce human rights-related barriers to HIV services by mainstreaming the lessons learned from the Breaking Down Barriers initiative across the Global Fund portfolio?

*Very important / Important / Moderately important / Slightly important / Unimportant*

95. Do you agree that investments are needed in societal enabling approaches that remove legal barriers, shift harmful social and gender norms, reduce inequalities and improve institutional and community structures?

*Strongly agree / Agree / Undecided / Disagree / Strongly disagree*

96. How important do you feel that the efforts of the Global Partnership are to bring different stakeholders together at country-level, led by government and civil society, to develop country plans to jointly tackle stigma?

*Very important / Important / Moderately important / Slightly important / Unimportant*

97. To what extent do you agree with the following statement?

Co-action across development sectors is essential to ensuring the success of the 3 S’s of the HIV response (i.e. Society-, System- and Service-enablers), which are intended to ensure countries can meet their HIV goals. of the response to HIV that ensure that HIV services are non-discriminatory and person-centred. These are critical to stigma reduction and achieving HIV goals.

*Strongly agree / Agree / Undecided / Disagree / Strongly disagree*

98. Do you agree that more evidence-based strategies need to be identified and be adequately funded by appropriate development agencies?

*Strongly agree / Agree / Undecided / Disagree / Strongly disagree*

99. Countries are at different stages in determining where to target investments in societal enabling approaches.

To what extent do you agree or disagree that ideally, countries should focus first on removing legal and societal barriers to HIV services to enable them to achieve the 95-95-95 targets?

*Strongly agree / Agree / Undecided / Disagree / Strongly disagree*

100. Do you feel that countries should commit to removing/updating laws that impede HIV services; and ensure that no more than 10% of people living with HIV and key populations experience stigma and discrimination?

*Yes / No / No opinion*
