# Supplementary material for: Consensus on addressing HIV-related stigma and achieving the societal enabler targets using an adapted Delphi process
Source: BMJ Open. 2025 Aug 12;15(8):e092516. doi: 10.1136/bmjopen-2024-092516 (PMC12352172; doi:10.1136/bmjopen-2024-092516)
Supplement: Supplementary file 2 [file bmjopen-2024-092516.reviewer_comments.pdf]

PEER REVIEW HISTORY

BMJ Open publishes all reviews undertaken for accepted manuscripts. Reviewers are asked to complete a checklist review form and are provided with free text boxes to elaborate on their assessment. These free text comments are reproduced below.

ARTICLE DETAILS

Title (Provisional)

Consensus on addressing HIV-related stigma and achieving the societal enabler targets using an adapted Delphi process

Authors

Molesworth, Kate; Nkosi, Sbongile; Camacho, Salvador; Caswell, Georgina; Salem, Simone; Baral, Stefan; Kamarulzaman, Adeeba; Sprague , Laurel; Stackpool-Moore, Lucy

---

VERSION 1 - REVIEW

**Reviewer**	**1**
**Name**	**Abdulai, Abdul-Fatawu**
**Affiliation**	**The University of British Columbia, School of Nursing**
**Date**	**24-Nov-2024**
**COI**	**None**

Many thanks for the opportunity to review this manuscript. Overall, the manuscript is sound, and the topic is quite important and timely. I have some observation related to the methods, and I am not sure these can be addressed at this point. If they can't be addressed, they should be acknowledged as limitations

• The authors stated that "The high-level of consensus emerging from the first round of questions led us to define a preliminary consensus of 98-100% of respondents agreeing on all degrees of agreement on the five-point Likert scales used". This means the consensus level was set after getting the results which is often not the case in Delphi studies. This means, the researchers were more or less controlling what should and should not be included. They should have set the consensus level before setting out on the first round of the Delphi.

• It also seems that the two rounds of Delphi were for two different purposes. Delphi study is when a set of items on a particular topic are sent to the same participants for at least twice. But it looks as if the first round was to obtain consensus regarding the evidence relating to HIV-related stigma while the second round was designed to build consensus on prioritizing action that the panel considered necessary. The are two different purposes I

doubt if that qualifies as a Delphi study. Its better to frame it as 2 separate set of surveys rather than a Delphi study.

• I think the authors needs to explain how the data was analyzed. A 5-point Likert scale was used so it would be good to explain what was considered as a consensus rather than just stating the percentage points.

• The authors also talked about majority of the respondents coming from civil society and I think it will be nice to provide examples of some of those civil society to see if they truly work in the HIV space.

• The consensus points look a bit problematic. I thought the purpose of the study was to ask participants to agree on specific examples of frameworks, programs, community leadership, intersectional stigma and discrimination ………. However, it looks like concrete examples of these thematic areas were not provided but rather the consensus points were pointing to the need or importance of this thematic areas.

**Reviewer**	**2**
**Name**	**Joanne , Csete**
**Affiliation**	**Columbia University, Populatino and Family Health**
**Date**	**25-Jan-2025**
**COI**	**I declare that I was among the Delphi respondents for this**
**study.**	

The article is well written and insightful and should be published. For those who put stock in Delphi processes it will be a significant addition to the literature. Just a few thoughts:

--The Breaking Down Barriers paragraph should be updated. Breaking Down Barriers did not end with the Global Fund strategy that ended in 2022. It was expanded to 24 countries in 2023-24. See https://www.theglobalfund.org/en/human-rights/breaking-down-barriers/

--It would be helpful for the authors to say more about anything new that emerged from this exercise since most of the consensus elements seem already to be highlighted in the materials of the Global Partnership, UNAIDS, etc. How exactly do these results contribute to new ideas about definitions or frameworks?

--It would be useful to say something about whether there is a strategy for using the "Call to Action" other than publishing it in this article. Who particularly would be helped by having these results and in what form?

--Almost not worth mentioning: the name of Joanne Csete is misspelled among the Delphi respondents and her title, adjunct associate professor, is not correctly stated.

VERSION 1 - AUTHOR RESPONSE

Reviewer: 1<br>Dr. Abdul-Fatawu Abdulai, The University of British Columbia<br>Comments to the Author:<br>Many thanks for the opportunity to review this manuscript. Overall, the manuscript is sound, and the topic is quite important and timely. I have some observation related to the methods, and I am not sure these can be addressed at this point. If they can't be addressed, they should be acknowledged as limitations	Thank you. Done.
The authors stated that "The high-level of consensus emerging from the first round of questions led us to define a preliminary consensus of 98-100% of respondents agreeing on all degrees of agreement on the five-point Likert scales used". This means the consensus level was set after getting the results which is often not the case in Delphi studies. This means, the researchers were more or less controlling what should and should not be included. They should have set the consensus level before setting out on the first round of the Delphi.	We have taken on board your comments and changed the terminology to "survey" and clarified that the methodology was an adaptation of Delphi methodology.<br><br>We have more fully set out our rationale for not pre-setting a percentage consensus threshold. This is set out in the Methods section on page 4.
It also seems that the two rounds of Delphi were for two different purposes. Delphi study is when a set of items on a particular topic are sent to the same participants for at least twice. But it looks as if the first round was to obtain consensus regarding the evidence relating to HIV-related stigma while the second round was designed to build consensus on prioritizing action that the panel considered necessary. The are two different purposes I doubt if that qualifies as a Delphi study. Its better to frame it as 2 separate set of surveys rather than a Delphi study.	Agreed. We have elaborated in the Methods section our rationale for adapting the Delphi-method to serve this purpose.<br>.<br><br>We have taken on board your comments and

	changed the terminology .
• I think the authors needs to explain how the data was analyzed. A 5-point Likert scale was used so it would be good to explain what was considered as a consensus rather than just stating the percentage points.	This is now further defined in the methods as well as the results sections. Additionally, the scale for each question is detailed in Annex 4.
• The authors also talked about majority of the respondents coming from civil society and I think it will be nice to provide examples of some of those civil society to see if they truly work in the HIV space.	The organisations and countries of respondents are detailed in "Annex 2 EXPERT PANEL MEMBERS". This is now also flagged in the text on page 6.
• The consensus points look a bit problematic. I thought the purpose of the study was to ask participants to agree on specific examples of frameworks, programs, community leadership, intersectional stigma and discrimination ………. However, it looks like concrete examples of these thematic areas were not provided but rather the consensus points were pointing to the need or importance of this thematic areas.	We have set out in the methods that in the first survey we presented evidence from the literature and sought consensus on the importance expert panellists attributed to this. This then formed the basis for the second survey regarding action on evidence.
Reviewer: 2<br>Dr. Joanne Csete, Columbia University<br>Comments to the Author:<br>The article is well written and insightful and should be published. For those who put stock in Delphi processes it will be a significant addition to the literature. Just a few thoughts:	--------------------
--The Breaking Down Barriers paragraph should be updated. Breaking Down Barriers did not end with the Global Fund strategy that ended in 2022. It was expanded to 24 countries in 2023-24.<br>See https://www.theglobalfund.org/en/human-rights/breaking-down-barriers/	Paragraph updated.
--It would be helpful for the authors to say more about anything new that emerged from this exercise since most of the consensus elements seem already to be highlighted in the materials of the Global Partnership, UNAIDS, etc. How exactly do these results contribute to new ideas about definitions or frameworks?	The contribution of this work was to seek consensus on priorities from existing statements (not create new ones). This has

	been brought out further in the text.
--It would be useful to say something about whether there is a strategy for using the "Call to Action" other than publishing it in this article. Who particularly would be helped by having these results and in what form?	Thank you – have added some details on this point.
--Almost not worth mentioning: the name of Joanne Csete is misspelled among the Delphi respondents and her title, adjunct associate professor, is not correctly stated.	Apologies! These errors now been corrected.
